# Clinical supervision in primary health care; experiences of district nurses as clinical supervisors - a qualitative study

**DOI:** 10.1186/s12912-015-0089-3

**Published:** 2015-07-28

**Authors:** Elisabeth Bos, Charlotte Silén, Päivi Kaila

**Affiliations:** Karolinska Institutet, Centre for Family Medicine, Department of Neurobiology, Care Sciences and Society, Alfred Nobels Allé 12, SE-141 83, Huddinge, Sweden; Karolinska Institutet, Department of Learning, Informatics, Management and Ethic, Stockholm, Sweden; Karolinska Institutet, Department of Neurobiology, Care Sciences and Society, Huddinge, Sweden

**Keywords:** District nurse, Supervisor, Primary health care, Qualitative content analysis

## Abstract

**Background:**

Learning in the clinical environment is an important part of nursing education. Several recent studies focusing on clinical learning have been based on hospital settings. Little is known about primary health care (PHC) as clinical environment where district nurses (DNs) or nurses supervise students. It is important to understand more about opportunities and difficulties in supervising in this area in order to develop PHC as an optimal learning environment for nursing students. The main objective of this study was to gain an understanding of supervisors’ experiences of supervising undergraduate students at PHC units.

**Methods:**

A qualitative research approach was used to collect data and analyse supervisors’ experiences. Six focus groups were carried out with 24 supervisors. Focus group data were audio-taped. The data were analysed using an inductive content analysis.

**Results:**

Three themes illustrated supervisors’ experiences: *abandonment*, *ambivalence* and *sharing the holistic approach*. Supervisors felt abandoned by their managers, colleagues and nurse teachers from universities. They experienced ambivalence due to simultaneously being supervisors for students and carrying out their daily work with patients. At the same time, they were proud to be DNs and willing to share their unique role to apply a holistic approach and continuity in patient care with students.

**Conclusion:**

When supervising students in PHC, social support and communication between supervisors and their colleagues and management as well as nurse teachers need to be taken into consideration both at universities and at primary health care units.

## Background

This study focused on DNs experiences of supervising nursing students during clinical placement in (PHC) units. Clinical placement is an essential part of nursing education allowing nursing students to develop their clinical competence. The clinical supervisor constitutes an important resource in this development and the relationship between the student and the supervisor influences how students learn nursing. Being a supervisor is becoming increasingly important because undergraduate students today learn more theory and spend less time in hands-on training in clinical practice than heretofore. Most studies of nurses as supervisors were conducted in hospital settings [1–3]. Knowledge about DNs’ experiences as supervisors within the PHC system is limited.

To work as a district nurses in PHC units differs from working in hospitals in many ways. Working in PHC traditionally includes preventive care, help to self-care and home health care. By working in patients’ homes, one must take patients’ autonomy, self-determination and choice of lifestyle into account [4, 5]. District nurses in Sweden are certified (registered) nurses with completed specialist education in primary health care. They provide nursing care in patients’ homes and at PHC centres in home health care, DNs often establish long-term and close relationships with patients and their families [5, 6]. This is in contrast to hospitals, where treatments and services are provided by a great number off medical and nursing staff working in teams. Reducing the length of a patient’s hospital stay in many acute hospitals [7].

Supervisors are responsible for supervising students’ in clinical placements. While the concept of supervisor might be called *mentors* or *preceptors*, among other titles [6, 8, 9], *supervisor* is widely used in many studies and refers to people who monitor and directly oversee students [10]. Supervision covers several pedagogical activities, which refer to the guidance and support of students in their learning process and assessment of students’ performance. Hilli et al. [11] indicated that in the beginning of a clinical placement supervisors more supportive working close to the students in different learning activities. When the student is ready to take more responsibility, step by step the supervisor tends to supportively stay in the background. The supervisors intend to facilitate students’ individual learning processes and professional development by encouraging students to reflect on nursing procedures and guiding students in communication skills and interaction with patients. Supervision also provide support on increasing students’ own responsibility to nurse patients independently [12].

On the other hand, some studies reported that not all clinical settings can create favourable learning environments for nursing students’ learning. For example some supervisors’ experience that they did not have enough information about nursing programmes. District nurses experienced being ill prepared for the supervision role due to insufficient communication with universities, and they could not assess students’ learning outcomes optimally [13].

The dynamics of the relationship between a student and a supervisor has been highlighted as an important aspect in students’ learning. Several studies of students’ experiences [3, 14] show that the mutual relationship between a student and an individual supervisor plays a key role in the clinical learning environment, [11, 15]. Supervision normally involves a supervisor and a supervisee (e.g. a nursing student) and in PHC one supervisor has primary responsibility for one student’s clinical learning [16]. A relationship between a supervisor and a student is based on trust and support for students to reflect on nursing skills and be professional [16–20]. Students value reflection with their supervisors since it helps students improve their patient care [21].

The shift into a higher nurse education setting and a broad academic profile has contributed to a very complex learning process. What students learn in the classroom bears little resemblance to what they experience in practice [22]. The clinical learning process aims to promote the integration of theory into clinical practice with the nurse teacher in the coordinator role [23]. This makes supervisors’ roles challenging and the quality of supervision depends on supervisors ability and experience to best support students´ learning process applying theoretical knowledge in practice manage the learning activities [24–26]. A former study of Gillespie and Fetridge [27] also concluded that it is necessary for nurse teachers to assume an active role in students’ learning processes in clinical settings.

According to the World Health Organization [28], primary health care have become an important part of the health care system in most countries, since an increasing number of patients receive care at home. However, the prerequisites for supervising in PHC have not been thoroughly investigated and knowledge about supervising nursing students in PHC is very limited. The research carried out about supervising students is mostly from hospital care settings [29]. This study aimed to gain understanding of supervisors’ experiences of supervising undergraduate students at primary health care units.

## Methods

### Design

A qualitative research approach was used to explore supervisors’ experiences. Data were collected using focus groups, which is a particular form of group interview. The method can inspire group dynamics in discussions among people of similar backgrounds [30]. The supervisors were selected for a group through purposive sampling. The data collection strategy was a semi-structured interview with guide questions. An inductive content-analysis method was used to analyse the data gathered during the sessions [31].

### Participants and settings

In Sweden, 20 county councils are responsible for organizing the PHC system. Each county council facilitates the provision of health and medical care for the inhabitants in the municipalities for which it has responsibility. All nursing students in Sweden spend part of their clinical education at PHC units with DNs as supervisors. The duration of the period in PHC and the term of the study programme varied from university to university. The study sample consisted of 24 DNs (23 women and one man) and one registered nurse who had previously worked as a DN. Participants worked at five PHC units (public and private) within the Stockholm County Council organization. Inclusion criteria were experience of working at the PHC unit and supervising undergraduate nursing students. All supervisors received information that the interviews would be recorded and transcribed. Interviews were conducted in a designated room at the PHC units and each lasted between 50 and 60 min.

### Data collection

Data were collected by means of six focus groups interviews. During the interviews, one of the authors acted as moderator and another as assistant moderator. The moderator followed the interview guide, a topic guide written in advance, which was a list of areas arising from previous literature and relating to their experiences of supervising nursing students. The areas were “Prepared for supervision”, “Organizing supervision”, “Relationship between student and supervisor” and “Collaboration and support”. Some follow -up questions were used to elicit more detailed information, e.g. “Explain what you mean by that.” The discussion was lively and the moderator tried to involve everyone in the interviews by presenting additional questions. A discussion ensued and all supervisors had opportunities to respond to questions.

### Data analysis

The inductive content analysis was made in the following stages. The authors:

1. Assigned an ID to each focus group and its PHC unit to link a specific set of notes or quotes with a group/unit.
2. Listened to all interview recordings to become familiar with each interview’s content.
3. Implemented several rounds of naïve reading.
4. Took notes on units of meaning relevant to the study’s aim.
5. Clustered units of meaning with the same content and condensed the units into subthemes
6. Extensively discussed themes and subthemes that emerged – organizing and reorganizing themes and subthemes until consensus was reached on the interpretation of the data [30] (Tables 1 and 2).

**Table 1:** Subthemes and themes from thematic content analysis

Subthemes	Themes
1. Insufficient dialogue and support from universities	*Abandonment*
2. Uninterested management and colleagues	
3. Students as burden or resource	*Ambivalence*
4. Security and insecurity	
5. Conflicts of loyalties	
6. Learning opportunities from complex PHC situations	*Sharing a holistic approach within the PHC system*
7. From dependence to independence	
8. Finding time for reflection

**Table 2:** Examples from the analysis of the first theme; *Abandonment*

Meaning units	Condensed	Subthemes
Group 6: Have not received enough support from universities in assessing students. It is universities’ responsibility to arrange students’ clinical placements.	Supervisors need support from universities.	Insufficient dialogue and support from universities.
Group 4: Briefly met with nursing teachers at networking meetings but no much-needed in-depth discussions about the clinical supervisor role took place.	No in-depth discussions with nursing teachers(regarding clinical supervisors’ role) take place at networking meetings.	" " "
Group 5: Lack of support from our bosses.	Manager provides no support.	Uninterested management and colleagues. " "
Group 6: Support from our colleagues insufficient or not forthcoming.

### Rigour

Trustworthiness criteria were used to evaluate rigour for this study [31]. Trustworthiness involves concepts such as dependability, credibility, confirmability and transferability. To ensure dependability, the authors described in detail any changes in data collection and proposed ways in which changes might affect results. They analysed the material independently to ensure credibility. They discussed whether or not more information was needed. Discussion of experiences and perceptions of the research topic before data collection facilitated the identification of inherent biases. To ensure confirmability the interviewer listened carefully to supervisors’ responses and then asked for clarification. To enhance transferability, the participants, context and process of analysis have been described with great care.

### Ethical considerations

The research ethics committee at the Karolinska Institute in Stockholm approved the study (2007/1531-31/3). All supervisors signed informed consents and were briefed on their right to withdraw from the study at any time. Their personal information was treated with total confidentiality. Transcripts were anonymized by not including any information about the focus group participants in a manner that identified them. During the analysis phase, only the group was given its own ID.

## Results

The average age of the supervisors was 51 and the average number of years of professional experience was 24 with a minimum of two years’ experience of supervising students. Only 10 of the 24 supervisors had special pedagogical training. Seven of the 24 supervisors held a BSc degree and of these three also held an MSc degree.

Three themes and eight subthemes emerged from the analysis, as shown in Table 1. Table 2 shows an example from the first theme of the analysis.

### Theme 1: Abandonment

Supervisors expressed feelings of abandonment and vulnerability in their role as supervisors. Three subthemes illustrated varying experiences of abandonment:

#### Insufficient dialogue and support from universities

The supervisors felt that universities did not support them sufficiently. For example, when problems arose, supervisors experienced difficulties to receive assistance and support from nurse teachers; they were left on their own:

We never see nurse teachers here; we feel alone (group 4). Unfortunately, no one from the university has been here, and we were given many practical tasks (group 2).

Supervisors communicated with students when placement periods began. They listened to students’ expectations and discussed their learning goals. They wanted to know more details about the students’ learning outcomes and learning activities during the placement. They did not have information about contact persons or nurse teachers who were responsible for the students’ clinical learning. Supervisors felt they were abandoned. They expressed frustration concerning insufficient information and dialogue, as the following statements illustrate.

A nurse teacher never contacted me … no contact with a teacher ever. I would like to ask the teachers about the placement’s objectives. I don’t even know the teachers’ names. I normally hear them from students and then contact the teachers via email (group 2). They have also changed the term in which students do their clinical practice, what should we consider? What is expected of me? I feel insecure (group 3).

I want more information about the curriculum and assessment method. I want to be prepared (group 1).

To identify what students are supposed to learn, supervisors hold discussions with students. Supervisors said that working with patients in PHC was not always compatible with students’ learning objectives. The following statements reflect supervisors’ frustrations.

I don’t understand what it means. It’s an educated guess [several supervisors nodded in approval], too little time here (group 3). It’s difficult to understand students’ expected learning outcomes. Sometimes, expectations differ from our work in primary health care (group 4).

Supervisors felt they would like to be more often involved in students’ education by regularly attending network meetings of supervisors and nurse teachers. But when such meetings occurred, many supervisors reported that they had found it difficult to hold in-depth discussions with nurse teachers.

Through these networking events I get to meet the nurse teacher, but we have so little time that there is no in-depth discussion about my role as supervisor (group 4).

Supervisors also expressed difficulties when supervising students who were not interested in nursing care or learning in PHC. They expressed feelings of loneliness without support from the universities:

There are students who are not involved or committed during their placements. They just sit in a chair and it’s not easy to supervise them (group 3).

#### Uninterested management and colleagues

The supervisors stated that they did not receive support from their unit, i.e. they did not receive adequate assistance from PHC management, who were often not interested in the students’ learning and did not allocate time for supervision. Supervisors expressed frustration about this:

It [the importance of supervising students] must come from the manager. The manager talking to a student? Never, no, no. (group 5).

Supervisors also felt they did not get help and support from their colleagues and this had consequences for the atmosphere at the unit.

Poor support from our own profession generates a bad atmosphere for students. There must be transparency in the profession, but now we have poor support within our own profession (group 6). We get no support from anywhere (group 5).

### Theme 2: Ambivalence

Supervisors’ experiences concerning supervision were characterized by ambivalence. They were for *and* against supervising students. They felt some reluctance to take on the assignment. Three subthemes illustrated ambivalence:

#### Students as burden or resource

Content that fitted into this subtheme reflected whether or not students were a burden. Supervisors described how they supervised students on a rotation basis. The supervisor whose turn had come to supervise students was responsible for one student during the placement. Supervisors were divided regarding whether or not supervision should be obligatory.

They must respect colleagues who don’t want to be supervisors (group 1). Some don’t like to supervise. Some workplaces actually say” no” to students. And right now, supervising students feels like a heavy burden (group 5).

For some DNs, supervising students was not a burden; on the contrary they were satisfied with this task. These supervisors stated that students gave them constructive feedback on their supervision and nursing care. They perceived this as positive and a help for further development of supervision. They described supervision as constant give-and-take between students and themselves, which could potentially lead to personal fulfilment:

Give and take all the time, it’s challenging to have students, it’s exciting to have students (group 5).

#### Security and insecurity

DNs were secure in their work as nurses – and not strangers to working independently within the PHC unit. But they expressed insecurity regarding the supervisor role. They felt insecure about how to assess students’ learning outcomes because they did not understand the learning assessment form and found students’ assessments difficult. Before assessment meetings with students, supervisors gathered information about their performance through discussions with their colleagues.

We don’t like the assessment stage. We have difficulty rating students’ performances on a scale from 1 to 10. A 2 or 3 points on this scale makes students feel bad (group1). I find it difficult to put an X on a scale item that is fair to the student (group 4).

#### Conflicts of loyalty

Self-reported conflicts between PHC units’ expectations and universities’ expectations fit into this subtheme. Supervisors stated that even students could notice this conflict and a supervisor’s heavy workload. When supervisors wanted to devote time to students, their workload often did not allow them to do so. They described the nature of their work as district nurses as busy and generally variable from day to day. This made it difficult to plan supervision of students. Consequently, supervisors felt torn between caring for patients and properly supervising students:

Oops, today I have time so then you [student] can come with me (group 2). It takes time with students, and I don’t have it (group 3). As supervisors, we have more responsibility now, and our jobs are stressful (group 4).

Despite experiencing conflicts between their nursing role and their supervision role, supervisors tried to create a welcoming, learning atmosphere for students. They wanted to be well-prepared before the students arrived – with the intention of giving students a good introduction.

### Theme 3: Sharing the holistic approach within PHC

Supervisors were proud of their profession and to work as a DN. They described DNs’ unique role of applying a holistic approach and continuity in patient care. DNs were willing to share their enthusiasm with the students so that they could better understand the complexity of and variety within the DNs’ work and profession. They wanted students to gain experience both in home health care and at PHC centres. Students thus receive a comprehensive and holistic understanding of nursing at the PHC unit.

#### Learning opportunities from complex nursing situations in PHC

*S*upervisors pinpointed differences between learning activities in hospitals and at PHC units. At the PHC units, students meet people of all ages and all social backgrounds with various and complex health problems and diseases. They also visit patients in their homes, where they might end up in very complex encounters.

Nursing in a holistic context is unique in the district nurse profession. Here they [students] will get the big picture (group 1). The district nurses’ work involves patients from a wide cross-section of society (group 6).

When students go on home visits, it is not only to find a disease. In primary health care, when you visit patients at home, you can see things such as a dirty home, which becomes your problem too. I think this is the big difference (group 1).

#### From dependence to independence

Findings *were* conflicted regarding supporting nursing students’ independence *as* [and] some supervisors were unwilling to support it. At the beginning of the learning period, the student and his/her supervisor make home visits to patients. Gradually, supervisors allowed students to take care of patients more independently and establish close relationships with patients and their families. The supervisors demonstrated trust in students’ abilities and believed it was valuable for student’s learning to spend time alone with patients and their families.

I always ensure that my students go alone on home visits. It’s usually constructive to see them grow (group 2).

However, some supervisors were cautious about leaving students alone with patients, because they did not know whether or not it was allowed and whether students had enough knowledge and skills to take care of patients. Supervisors felt that they have the ultimate responsibility for patient care:

I did not know they could to do that; I think it is a responsibility issue. I dare not let my students be alone during home visits. You never know what can happen (group 4).

#### Finding time for reflection

Supervisors thought that a way for students to learn patient care holistically was to reflect on complex nursing situations, for example after a home visit when students had been exposed to a patient’s unhealthy lifestyle. Because of workload it was difficult to find time for reflection. Supervisors were often innovative and found solutions to the dilemma.

If there is difficulty in finding time for reflection, we try at the end of the day or between home visits (group 3).

During a walk between home visits, we take time to discuss things (group 2).

## Discussion

The results from this study show that supervisors encounter both favourable and unfavourable conditions in supervising. This entailed opportunities for and challenges to supervision within the PHC unit. Overall, the supervisors lacked information about students’ clinical education; they demanded better support and cooperation from nursing education stakeholders. Results illustrated by the two themes – *abandonment* and *ambivalence* – underscored feelings from supervising students within the PHC unit and demonstrated how exposed supervisors were in this situation. The first theme reflects supervisors’ experiences around the lack of contact with the university, and second theme theirs ambivalence about the assignment to supervise students.

The third theme – *sharing the holistic approach in PHC* – showed supervisors’ positive experiences of working within the unit and emphasized the favourable learning conditions that they can and want to offer students. This third theme reflects the opportunities to capture the whole picture of patient care in PHC.

### Theme 1: Abandonment

The study identified unfavourable conditions on individual and unit levels. Abandoment was one of the main themes in this study and supervisors felt that universities abandoned them. Poor communication between nurse teachers and supervisors about what is expected of the supervisor creates feelings of abandonment in the supervising situations. The importance of clear and open communication on the role and the function as a supervisor has been reported earlier [32]. Nurse teachers from universities are responsible for providing supervisors with actual and adequate information on changes in programmes and learning outcomes. Unclear communication between nurse teachers and supervisors was consistent with findings from earlier studies of supervisors’ experiences of supervising nursing students [16, 33]. One explanation on poor communication might be that PHC units are normally geographically widespread, which may complicate collaboration with universities. Also heavy workload in PHC demands on supervisors’ time and priority in patient care and this can affect to poor communication with universities.

To facilitate communication, the nurse teachers organize networking meetings but supervisors in this study felt that these meetings were not enough. Supervisors themselves seemed to be passive; they did not actively seek information on their own. Previous studies reported that it is important for supervisors to actively seek up-to-date knowledge to prepare them for their supervising role [34, 35]. The results from this study were fully in line with earlier studies that show that supervisors must be more closely involved with students’ learning activities and academic faculties’ goals, objectives and expected outcomes [2, 36].

Supervisors in this study also felt that the PHC units’ management *abandoned* them. Receiving feedback on the function as a supervisor from units’ manager has been shown to be important [32]. This finding agrees with other studies that have shown the importance of managers’ involvement in supervision, so that supervisors can feel appreciated and in turn create good student learning conditions [2, 37, 38]. Management’s attitude might influence supervisors’ attitude toward supervision and supervisors’ abilities to supervise students. Supervisors require much more management support in their supervisory roles [12, 24].

### Theme 2: Ambivalence

Initially, the supervisors expressed many varying feelings about supervising students. Supervisors did not see supervising as part of their ordinary work, but rather as an additional task to perform. They showed some uncertainty about how to supervise students; supervising was not clearly defined or it was not aligned with universities’ expectations.

One interpretation of supervisors’ unpleasant experiences of supervising students might be a combination of PHC units’ requirements for good nursing for patients, increasing workload and vague, unstructured directives from universities. A barrier to successful supervising was insufficient commitment when it comes to sharing supervising responsibility with colleagues. Support from colleagues can provide an encouraging, inspiring learning environment for students and make supervision easier for all stakeholders [39].

In this study, all supervisors monitored students on a rotation basis. It was assumed that everyone was obliged to take a student during the term – which was also shown in a previous study [16]. In addition, they should supervise students without prior discussions or agreement on whether or not certain DNs were willing or had the educational competence to supervise students. Warne et al. [24] showed in their study that supervisors who create a friendly atmosphere which is warm and welcoming and show a friendly attitude towards supervision promote students’ learning. Hilli et al. [11] also find that students who feel secure in the relationship with their supervisor dared to ask questions and reflect more on learning activities.

Most supervisors in this study became qualified before 2007 when the Bologna education system was implemented in Sweden [23]. Students’ current educational goals are different from those of previous years and students often require qualified support to integrate theory into practice [40]. This may be an explanation why supervisors had difficulty to understand the assessment form and they had insufficient knowledge of how to successfully supervise students. The need for support in student performance assessment has been addressed in other studies that report that supervisors who had insufficient theoretical knowledge about nursing could not help students to reach their desired learning goals [41]. There thus seems to be a gap between theoretical education and practical training even in the context of PHC as a clinical learning environment. This theory-practical gap is one of the major challenges in nursing education [42, 43]. Note that only 7 of 24 supervisors interviewed had a BSc degree - hence there is need for more support for these supervisors.

Indeed, there is a need for more communication and collaboration between PHC units and universities regarding students’ learning and supervising. Research needs to identity the connection between students’ learning outcomes and the possibilities to achieve them in PHC.

### Theme 3: Sharing the holistic approach within PHC

Supervisors in this study had extensive experience of supervising students and felt well-acquainted with their required tasks. Supervisors expressed pride in being DNs and wanted to show students what a holistic approach to patient care means when working as a DN. They emphasized the special nature of home care compared to hospital care. Supervisors were committed to involving students in complex and holistic patient care by supporting them in making home visits independently. This creates both opportunities for and challenges to students’ learning how to take responsibility and become an independent professional [18, 20]. In this study the DNs let the student often go alone to patients home, precisely in order to allow for students independent learning. Consequently, supervisors and students can reflect and share similar experiences of patient care. Students can contribute new ideas that can stimulate discussion with supervisors [44].

Supervisors in this study wanted to prepare as much as possible to give students a good introduction. Building confident relationships with students seemed to be a way for the supervisors to reduce their feelings of abandonment in supervision; this can even dispel feelings of loneliness. We previously showed [14] that the supervisors in PHC have an important role, in creating a pedagogical atmosphere for student’s learning.

Good supervisor preparation is crucial for supervision, since it enhances learning and helps reduce their feeling of uncertainty [45]. Supervisors tried to understand and internalize the students’ learning objectives by participating in network meetings about supervising. This might be one way that supervising can be more efficient [24].

### Limitations

Qualitative studies are limited to a local context but provide a deeper analysis of that context. It is therefore important to give a detailed description of the context used in this study so that it may be generalized to similar contexts. One interviewer supervised nursing students at a PHC unit and had knowledge about this particular context, which facilitated the interviews but might lead to some information being taken for granted and this might influence the study’s results. The focus groups were small and the experiences expressed are limited to the groups. To ensure trustworthiness, i.e. how well data processing meets the aim of the study, how the interviews were conducted was therefore important [30]. The extent of the research was limited to a small part of Sweden’s PHC system and no general conclusions can therefore be drawn.

## Conclusions

These findings shed light on the complexity of the supervisor role within the PHC system and conditions that may promote or limit good learning environments. The findings emphasize the fact that DNs/supervisors within the PHC system feel abandoned and lack support, particularly from their management, when they try to resolve conflicting goals between the PHC system and universities. Not all DNs are adequately prepared to undertake the supervisor role and consequently they cannot optimally supervise nursing students. A closer, more systematic collaboration between care providers and universities is necessary to improve nursing students’ learning conditions in PHC settings. There is therefore a need for more studies in PHC contexts to allow greater understanding of conditions for supervision.
